# Implementation of health-promoting retail initiatives in the Healthier Choices in Supermarkets Study—qualitative perspectives from a feasibility study

**DOI:** 10.1186/s12916-024-03561-2

**Published:** 2024-09-02

**Authors:** Katrine Sidenius Duus, Tine Tjørnhøj-Thomsen, Rikke Fredenslund Krølner

**Affiliations:** The National Institute of Public Health, University of Southern Denmark, Copenhagen, Denmark

**Keywords:** Implementation, Qualitative research, Health promotion, Supermarkets, Involvement, Intervention

## Abstract

**Background:**

Improving food environments like supermarkets has the potential to affect customers’ health positively. Scholars suggest researchers and retailers collaborate closely on implementing and testing such health-promoting interventions, but knowledge of the implementation of such interventions is limited. We explore the implementation of four health-promoting food retail initiatives selected and developed by a partnership between a research institution, a large retail group, and a non-governmental organisation.

**Methods:**

The four initiatives included downsizing of bags for pick’n’ mix sweets and soda bottles at the check-out registers, shelf tags promoting healthier breakfast cereal options, and replacing a complimentary bun with a banana offered to children. The initiatives were implemented for 6 weeks (or longer if the store manager allowed it) in one store in Copenhagen, Denmark. Data were collected through observations, informal interviews with customers, and semi-structured interviews with retailers. We conducted a thematic analysis of transcripts and field notes inspired by process evaluation concepts and included quantitative summaries of selected data.

**Results:**

Two out of four initiatives were not implemented as intended. The implementation was delayed due to delivery issues, which also resulted in soda bottles not being downsized as intended. The maintenance of the shelf tags decreased over time. Retailers expressed different levels of acceptability towards the initiatives, with a preference for the complimentary banana for children. This was also the only initiative noticed by customers with both positive and negative responses. Barriers and facilitators of implementation fell into three themes: Health is not the number one priority, general capacity of retailers, and influence of customers and other stakeholders on store operation.

**Conclusions:**

The retailers’ interests, priorities, and general capacity influenced the initiative implementation. Retailers’ acceptability of the initiatives was mixed despite their involvement in the pre-intervention phase. Our study also suggests that customer responses towards health-promoting initiatives, as well as cooperation with suppliers and manufacturers in the development phase, may be determining to successful implementation. Future studies should explore strategies to facilitate implementation, which can be applied prior to and during the intervention.

**Supplementary Information:**

The online version contains supplementary material available at 10.1186/s12916-024-03561-2.

## Background

What we eat affects our health and well-being [1]. Diet is associated with obesity, cancers [2], and mental well-being [3], and a healthy diet has been associated with lower all-cause mortality [4]. One important factor in improving diet is to create a food environment that supports a healthy diet [5, 6]. In modern societies, such as Denmark, supermarkets are the main source of food [7]. Supermarkets therefore hold a significant influence on what food we buy and potentially also eat [7–9]. Studies report associations between the concentration of supermarkets and overweight and obesity in the neighbourhood [10] and between the healthfulness of supermarkets and people’s diets [11, 12]. Moreover, unhealthy food and beverage products are promoted more often than healthy products and beverages in, for example, supermarkets [9, 13, 14]. This indicates a need to explore how and if it is possible to implement health promotion initiatives in supermarkets and whether customers respond to such initiatives as intended.

Studies show that health-promoting interventions in supermarkets can affect customers to purchase more healthy products [7, 9, 15–17]. Reviews and a meta-analysis have concluded that the most effective initiative in supermarket settings is price changes—the evidence points to the positive effects of reduced prices to increase the purchase of healthier products, especially fruit and vegetables [7, 17]. Even though price reductions seem to be effective, they seem more challenging to implement due to retailers’ drive for profit and low preference for financing such price cuts [7, 18]. There is some evidence that nudges in terms of product information and positioning, as well as altering the number of available products, can impact what products are being purchased [15, 16]. However, the quality of this evidence is low. Overall, most of the studies that have explored the effect of interventions in supermarkets have been conducted in the USA and other high-income countries [15, 16], in controlled settings, or applied a weak study design, such as non-randomised studies [16, 17]. To our knowledge, only a few studies have been conducted in Denmark [19–25]. These studies represent different designs and types of interventions: reformulation of private-label products to reduce calorie content [24], informational claims to promote low-salt foods [23], nudges via signs to promote sales of fruit and vegetables [22], positioning (shelf-space management) of dairy products [20], replacement of sugar confectionery with fruit and healthy snacks at the checkout [19], discount on fruit and vegetables combined with space management [25] and structural changes in supermarkets and education of supermarket employees as part of a multicomponent intervention [21] (the three latter studies are reporting from the same project). All but one study [23] found an effect of the applied intervention strategies, although mostly small or modest. This calls for more studies in real-life settings and investigations of why some interventions have the desired effect while others do not. Lack of effect may be explained by 1) customers not noticing or finding the initiatives relevant [19, 23], 2) customers buying other products instead of or additionally to promoted intervention products [20, 24], 3) the shelf organising effect [20], or 4) theory fail in regards to customer behaviour [22].

Several studies have explored facilitators and barriers to the implementation of health-promoting interventions in supermarkets. Reviews show that implementation is supported if the retailer is receptive to innovation, feels responsible for community health, and receives financial support or subsidies [26]. Furthermore, implementation is supported if the intervention provides the retailers with knowledge of health promotion and business skills [26, 27]. Other facilitators include compatibility with context and customers’ needs, positive customer responses to the initiative, the prospect of improved public image, establishment of partnerships, low retailer effort requirements, and increased profit or sales [26, 27]. Health-promoting interventions in supermarkets are hindered by high customer demand for unhealthy products and lower demand for healthy products, constraints of store infrastructure, challenges in product supply, high staff turnover, and lack of time [26, 27]. Other barriers are doubt regarding changing customers’ behaviour, poor communication between collaborators [26], high running costs, and risk of spoilage [26, 27].

Middle et al. [26] conclude that the underlying mechanism of barriers and facilitators of implementation is the (mis)alignment of retailers’ and intervention researchers’ interests. The authors, therefore, suggest a close collaboration between intervention researchers and retailers to work towards an alignment of interests and resolving or avoiding misalignment, which is supported by Gupta et al. [27]. However, knowledge of how such collaborative efforts affect the implementation of healthy food retail interventions is warranted.

The aim of this study is to explore the implementation, acceptability, and feasibility of four different health-promoting food retail initiatives to increase customers’ purchase of healthy food and beverages, which were selected and developed together with food retailers: 1) Promotion of healthier breakfast cereals and products using shelf tags, 2) downsizing of sodas sold at the checkout desks, 3) downsizing of bags for the pick’n’ mix sweets, 4) replacement of a complimentary bun for children with a banana. The study has three research objectives:

(I) To document the implementation and sustainment of the initiatives over time
(II) To explore the retailers’ and customers’ responses to and acceptability of the initiatives
(III) To investigate barriers and facilitators of implementation and sustainment of the initiatives.

## Methods

### Setting and the initiatives

This study was conducted in Denmark during 2020 and 2021, 2 years that involved two major societal events, first the coronavirus disease pandemic and later the start of the Russia-Ukraine war. Both events heavily influenced the circumstances of everyday life including opportunities for conducting research and running businesses. The specific influences on this study will be unfolded later in the findings and discussion sections.

In this study, we collaborated with the retailer Salling Group, which holds 34.2% of the market share of grocery retailers in Denmark [28]. Salling Group is owned by the Salling Foundations and has no shareholders—all profits go to reinvestment in the business and donations to sports (amateur and professional), charity, education, and research. Salling Group owns three national supermarket chains: føtex, Netto and Bilka, alongside other businesses. For the feasibility test, we collaborated with føtex, which owns over 100 stores all over Denmark, including 23 stores called føtex food. føtex (except føtex food) offers both groceries and many different non-food products (e.g. textiles, cosmetics, toys, electronics, and home accessories).

The initiatives were selected and developed by a partnership, including a group of researchers at the National Institute of Public Health, University of Southern Denmark, consultants from the Danish Cancer Society, and employees at the Corporate Social Responsibility (CSR) department in Salling Group, the marketing department at føtex, and two store managers (hereafter referred collectively to as ‘the retailers’) over approximately 2 years. The process involved in-person meetings, desk research (the use of existing material [29]), visits to the test store, and a prototype test of three suggested initiatives. The researchers initiated the collaboration and were responsible for designing the research study and data collection and analyses. The retailers hosted the site of the feasibility test, contributed to the selection and development of initiatives and co-managed the practical part of the study. The Danish Cancer Society was recruited by the research project to develop the initiatives. A detailed description of the collaboration and development process is reported elsewhere (Duus et al. *unpublished*).

The feasibility test ended up including four initiatives: 1) Promotion of healthier breakfast cereals and products using shelf tags, 2) downsizing of soda sold at the checkout desks, 3) downsizing of bags for the pick’n’ mix sweets, 4) replacement of a complimentary bun for children with a banana (suggested by the retailers). The initiatives were based on a compromise between the willingness of the retailers and the interest and ideas of the remaining partners rather than on what the literature suggests are the most effective strategies (Duus et al. *unpublished*). Detailed descriptions of the initiatives and the rationale behind them are found in Table 1.

**Table 1:** The four initiatives included in the Healthier Choices in Supermarkets Study

The area	The initiative	Assumptions of customer behaviour	Assumptions of health behaviour
Sodas	Downsizing of Coca-Cola original and Coca-Cola zero bottles from 500 to 375 ml in the coolers at the checkout desks and the cooler at the bakery^*^	Customers will continue to buy sodas from the coolers at the checkout desk and the bakery (impulse buys), and then naturally get a smaller volume due to the smaller-sized bottle (that customers might not notice)	Customers will consume fewer sodas, which will decrease their sugar and energy intake from sugar-sweetened variants and reduce other potential health risks of consuming beverages with sugar or artificial sweeteners (e.g. dental erosions)
Breakfast	Highlighting breakfast cereals by adding a shelf trailer on the price tag with the front of the package labelling ‘Keyhole’ and/or ‘Whole grain logo’ which indicate products with a high content of wholegrain and a low content of sugar, salt, and fat	The use of familiar labelling to customers will promote sales of healthier cereal products and inspire customers to buy a healthier alternative	The amount of sugar, salt, and fat from cereal products in the diet would be reduced, and the amount of wholegrain would increase
Candy	Downsizing of the pick’n’ mix bags for sweets by making them narrower	A smaller, narrower bag (that customers would not notice), that can contain fewer sweets and make the amount in the bag seem like more than the original bag, will lead customers to buy fewer sweets	The amount of sugar, fat and energy from candy in the diet is reduced
Fruit	Replacement of the complementary bun for children with a banana	Promote free fruit as a customer care initiative to accommodate those wanting a healthier alternative	The banana is thought of as a possibly healthier alternative to the bun (by the retailer). The emphasis on customer care is greater than health

^*^Both variants were included in the initiative to secure adherence to the downsizing working mechanism. Different sizes would increase the chance of substitution, e.g. choosing the larger variant if it was presented next to the smaller variant

The prototype test showed that 1) It was important to have a sign informing the customers about the initiative that offered a free banana to children instead of the usual free bun to create a better understanding of the changed offer; 2) Promotional shelf tags needed weekly maintenance as some would fall off; 3) It was difficult to sustain an initiative promoting ready-to-serve salads and ready-to-cook vegetables next to different fresh meats, as it met resistance among the staff due to being an additional task and led to more product waste (Customers did not expect to find these products next to the meat and therefore might not notice them). The learnings from the prototype test led to modifications of the implementation plan and the discard of the latter initiative. The prototype test also made us aware of how quickly the selection of food offered and the layout of the store changed over time, which the researcher, therefore, paid extra attention to during subsequent data collection. Moreover, the researcher made sure to update the list of products that should have a shelf tag a few weeks before the implementation to include new products offered.

The føtex marketing department developed a script to inform the staff at the test store about the feasibility test, explaining and showing each initiative and the aim of the study overall. This was sent to the store manager after being reviewed by the researchers. The store manager was responsible for informing all relevant staff about the implementation and maintenance of the initiatives. The føtex marketing department also made sure to inform the relevant suppliers. Employees at the test store and brand staff from a brewery (who stock the coolers at the check-out desks) implemented the initiatives in the store. The research group did not correct or maintain the initiatives in the store after they were launched; however, the researchers monitored it and reported back to the retailers, either at meetings or by email.

### Overall study design

The four initiatives were implemented in the test store for 6 weeks (or longer if the store manager allowed it) starting in September 2021. A føtex store in central Copenhagen (the capital city of Denmark) was chosen as the test store. This decision was made for pragmatic reasons, as the research institute is based in Copenhagen, and based on Salling Group’s decision as it offered their new store layout, which all stores were in the process of being converted to (it was the same store as where the prototype test was conducted).

We designed a qualitative study involving participant observations and interviews to evaluate the feasibility of the initiatives. The methods were designed to explore the partnership and collaboration (the aim of another publication [Duus et al. *Unpublished*]), as well as the implementation of the initiatives [30]. In the design of this study, we were inspired by McGill et al.'s (2020) two-phase framework of qualitative process evaluation from a complex systems perspective. This framework suggests an evaluation that looks at changes over time, starting with phase 1, the static system description and hypothesis generation about how the system might change when the intervention is introduced, followed by phase 2, an adaptive evaluation approach to the system undergoing change which follows emerging findings [31].

### Data collection

#### In-store observations

During October and November 2020, we mapped the store layout and customer flow in the test store as part of the static system description. Over 3 weeks, three research assistants performed 12 participant observations of 1005 min in total. The observations followed an observation guide which covered 1) the physical setting (e.g. the layout, placement of products, signs, and pictures); 2) the people (e.g. who are the customers? Are people shopping alone or together with others? How do they move around the store? What are the staff doing?) and 3) short interviews with customers (if possible) about their shopping at the particular store, and their thoughts about the layout of the store. The research teams’ access to the store was approved by the store manager, and research assistants wore a key chain with a sign showing their name and affiliation during the observations. During this data collection period, it was made mandatory to use face masks in supermarkets due to the coronavirus disease pandemic. As the implementation was delayed to approximately 1 year after this static description was completed, one participant observation in the test store was performed at the end of August 2021, just before initiative implementation, to document any major changes in the store layout and selection. Key lessons from these observations about the test supermarket and customers’ behaviour in the store included knowledge on ﻿1) the route around the store, 2) the different times spent at the store, 3) interactions with objects (e.g. products and phones), 4) interactions with children, 5) behaviour of the staff, and 6) sensory impression (Additional file 1). These lessons informed our following data generation and assisted in contextualising our analysis.

The first author monitored the implementation process through participant observations of status meetings (*n* = 2) and correspondence via email and phone with the store manager and the contact person at føtex. In-store participant observations were conducted during and after the feasibility test period, September 2021–May 2022 (*n* = 25 ~ 1795 min in total; see Additional file 2). These observations focused on documenting the presence of the initiatives as well as customers’ and staff’s responses to the initiatives. Access to the store was once again approved by the store manager, and the researcher wore a key chain. During the participant observations in-store, we conducted informal interviews with customers (see Additional file 2 for examples of questions), which lasted a maximum of 5 min each. The first author would approach people and ask if they were interested in answering a brief question. She introduced herself by her first name, where she worked and explained she was doing a research project about shopping patterns. The participant observations were documented by taking notes and photos. Handwritten notes were digitalised and written down at the first chance after leaving the store.

#### Qualitative interviews

Between November 2021 and February 2023, the first author conducted four semi-structured interviews with retailers (*n* = 3) who had been involved in the study (Table 2) to explore their views on the initiatives and the implementation process. Interview guides were used in all interviews alongside different prompts (e.g. timelines and documents). Interview guides were tailored to each participant’s specific role and involvement in the development and implementation of the initiatives. Besides questions related to the initiatives and the implementation effort, the guides included questions about the informants’ background and motivation for the project (personally and professionally), their view on their role and scope for action (individually and organisationally) and their perception of the collaboration with the other organisations. After the participants’ consent was given verbally right before the interview, the interviews were recorded and later transcribed verbatim.

**Table 2:** Interviews

Informant	Period and type of involvement	Time and place	Duration
føtex representative (A)	Development of the initiatives and decision-making at the executive level, 2019–2021	February 2023, telephone	40 min
føtex representative (B)	Primary contact at group/chain level and co-managing implementation, 2021	November 2021, online	50 min
Store manager	Primary contact in the test store, 2021	February 2022, online May 2022, online	55 min 55 min

### Analyses

To explore the level of implementation (research objective I), all field notes and photos taken during and after the feasibility test were reviewed to assess whether the initiatives were present and to what degree (e.g. x out of x possible tags).

To explore the perception of the initiatives among employees and customers (research objective II) and identify barriers and facilitators for implementing the initiatives (research objective III), we followed a thematic analysis inspired by Braun and Clarke [32]. Firstly, field notes and interview transcripts were read thoroughly and openly coded, by writing keywords in the margin of the material, with a focus on the two research objectives. After initial coding, the codes were summarised into broader themes, by writing them into a document with short descriptions and revised according to data excerpts and the full empirical material. The themes drew on the process evaluation concepts: acceptability, responsiveness [30], motivation, general capacity to implement [33] and commercial viability [34]. Lastly, the themes were named, and the final analysis was written up.

## Findings

We have structured the presentation of study findings as follows: Firstly, we present the implementation of the initiatives overall. Secondly, we present the implementation of each initiative, customers’ responses to them, and the retailers’ perspectives. Lastly, we present the overall facilitators and barriers to the implementation of the initiatives.

### Implementation of the initiatives

The implementation of the initiatives was challenged. Firstly, we found that not all the preparations for the implementation were finished in time for the scheduled day. On the scheduled day, the retailer decided to push back the implementation by 1 week. The main reasons were that there had been some misunderstandings around the ordering of the smaller sodas. It was informed that the smaller soda would be a 330 ml can instead of the 375 ml bottle at the price of DKK 10.00 (~ 1.3 euros). The 500 ml bottle usually sold at the coolers cost DKK 16.00 (~ 2.2 euros). The Danish Cancer Society and the research group had two concerns about this: 1) the use of a can instead of a bottle would make the interpretation of the results very difficult, as the bottle and the can have two different functions to the customer—with the can, the product would be consumed all at once, whereas the bottle with the screw lid could be saved for later after it had been open; 2) the price was too low—the price per litre would be lower on the smaller sodas than it had been on those replaced. No changes were made despite these concerns.

Secondly, just days before the implementation, the retailers informed the other partners that they would stick with cans for the test of smaller-sized sodas and that they would now be 250 ml. They acknowledged that both the size and the packing were not optimal but that the optimal 375 ml in a bottle was just not possible. Additionally, they informed the researchers that they could no longer find the new bags produced for the pick’n’mix sweet display.

These challenges led to a delay of the implementation of the initiatives by 1 week, but also a staggered implementation, where the initiatives were implemented when ready (the soda initiative 2 weeks later and the bags for pick’n’ mix sweets 8 weeks later). The retailers agreed to push back the end day correspondingly, upholding the 6 weeks of implementation. Table 3 shows an overview of the implementation of the four initiatives according to the day and week of the feasibility test period.

**Table 3:** Implementation of the four initiatives according to the day and week of the feasibility test period

Day and week of feasibility test period		Sodas at the coolers	Tags at the cereals *n* (%), *N* = 31	Bananas	Bags for pick’n’ mix sweets
Week 1	1	*Only 250 ml cans at the checkout desk* *Only 500 ml bottles at the bakery*	28 (90%) 1 misplaced	Bananas present Sign present	*N/A*
	3	*Only 250 ml cans at the checkout desk* *Only 500 ml bottles at the bakery*	28 (90%) 1 hidden 1 misplaced	Bananas present Sign not present	*N/A*
	6	*Only 250 ml cans at the checkout desk* *Only 500 ml bottles at the bakery*	28 (90%) 1 misplaced	Bananas present Sign present	*N/A*
Week 2	8	*Only 250 ml cans at the checkout desk* *Only 500 ml bottles at the bakery*	28 (90%) 1 misplaced	N/A	*N/A*
	11	*Only 250 ml cans at the checkout desk* *Only 500 ml bottles at the bakery*	28 (90%) 1 misplaced	Bananas present Sign present	*N/A*
	13	*Only 250 ml cans at the checkout desk* *Only 500 ml bottles at the bakery*	28 (90%) 1 misplaced	Bananas present Sign present	*N/A*
Week 3	15 (Official first day with sodas)	Only 250 ml cans in all coolers and fridges	28 (90%) 1 misplaced	Bananas present Sign present	*N/A*
	21	Only 250 ml cans in all coolers and fridges	28 (90%) 1 misplaced 1 tagged cereal sold out	Bananas present Sign present	*N/A*
Week 4	28	Only 250 ml cans in all coolers and fridges	27 (87%) 1 misplaced	Bananas present Sign present	*N/A*
Week 5	30	Both 250 ml and 330 ml cans in all coolers and fridges	26 (84%) 1 misplaced 1 tagged product sold out	Bananas present Sign present	*N/A*
	33	Only 330 ml cans at the check-out desk. Both 250 ml and 330 ml cans at the bakery	26 (84%) 1 misplaced 1 tagged product sold out	Bananas present Sign present	*N/A*
Week 6	36	250 ml cans in all coolers and fridges. A few 330 ml cans at the checkout desks	24 (77%) 1 misplaced	Bananas present Sign present	*N/A*
	39	Only 250 ml cans in all coolers and fridges	26 (84%) 1 misplaced	Bananas present Sign present	*N/A*
	41	Only 250 ml cans in all coolers and fridges	25 (81%)	Bananas present Sign present	*N/A*
	42 (Last day banana + cereal)	Only 250 ml cans in all coolers and fridges	23 (74%)	Bananas present Sign present	*N/A*
Week 7	43	Only 250 ml cans in all coolers and fridges	*23 (74%)*	*Bananas present* *Sign present*	*N/A*
Week 8	54	Only 250 ml cans in all coolers and fridges	*21 (68%)*	*Bananas present* *Sign present*	*N/A*
	56 (Last day sodas)	Only 250 ml cans in all coolers and fridges	*21 (68%)*	*Bananas present* *Sign present*	*N/A*
Week 9	57	*Only 250 ml cans in all coolers and fridges*	*21 (68%)*	*Bananas present* *Sign present*	*N/A*
Week 10	70 (Bags implemented six days before)	*Only 250 ml cans in all coolers and fridges*	*12 (39%)*	*Bananas present* *Sign present*	Only smaller bags are present
Week 11	76	*Only 250 ml cans in all coolers and fridges*	*12 (39%)*	*Bananas present* *Sign present*	Old larger bags are hung in front of the new smaller bags
Week 13	86	*Only 250 ml cans in all coolers and fridges*	*N/A*	*Bananas present* *Sign present*	Only smaller bags are present
Week 14	97	*Only 250 ml cans in all coolers and fridges*	*7 (23%)*	*Bananas present* *Sign present*	Only smaller bags are present (looks messy)
Week 15	99	*Only 250 ml cans in all coolers and fridges*	*6 (19%)*	*Bananas present* *Sign present*	Only smaller bags are present
2 weeks post-feasibility-test period		*Only 250 ml cans in all coolers and fridges*	*6 (19%)*	*Bananas present* *Sign present*	*Old larger bags are hung in front of the new smaller bags*
4½ months post-feasibility-test period		*No 250 ml cans in any of the coolers or fridges*	*3 (10%)*	*No bananas or signs are present*	*Old larger bags are hung in front of the new smaller bags*

*Italic font*  periods outside the implementation period of the specific initiative

### Smaller product sizes of sodas at the checkout desk

As seen from Table 3, we did observe the implementation of a smaller product size of the targeted sodas in all coolers, besides the one at the bakery, in the week leading up to the agreed date. We hereafter observed a full implementation of 250 ml cans during the first 2 weeks of implementation. During the third week and the beginning of the fourth week, we observed a mix of 250 and 330 ml cans or only 330 ml cans. The store manager explained that this was probably due to non-delivering from the supplier. At the end of the fourth week and for the last 2 weeks, we observed a full implementation of 250 ml cans. As the targeted size of the initiative was a 375 ml bottle, the initiative was not implemented as intended. After the 6-week feasibility test period, we observed that the smaller 250 ml cans were available in all coolers for at least eight more weeks. As expected, the presentation of the coolers fluctuated over the period. On days of stocking (Monday, Wednesday, and Friday), the coolers would look neat and full, while they would appear more empty or messy on other days.

#### Customer responsiveness

We observed very few customers who bought any products from the coolers, and we did not get to talk to any customers about the initiative. However, the observations in the store showed no distinct change in customers’ behaviour around the coolers nor expressions of discontent or excitement with the initiative. In an interview with the store manager, he explained that he believed customers had not noticed the change.

#### Retailer perspectives

The store manager was positive about the initiative, but from his perspective, the decision to implement it should be made at the procurement level and by the suppliers. However, he did have an opinion on how to implement it. The price needed to be fair according to the product it replaced. Moreover, he drew attention to the fact that it was the supplier’s personnel who stocked the products rather than his own. The store manager was, therefore, not surprised that the employees at the store had little to say about the initiative. føtex’s representative (B) was also positive about the initiative and expressed in the interview that the chain would be willing to implement it—if they found it to be the ‘right thing’ to do. However, the representative also emphasised the importance of agreeing with the suppliers, which is a time-consuming process and ‘not done in just six months’.

### Shelf tags for breakfast cereal products

From the first day of the implementation, some tags were missing, and one tag was consistently misplaced (Table 3). During the first 3 weeks, 10% (*n* = 3) of the tags were missing. This portion progressively increased to 23% until the end of the fifth week. In the sixth week, the portion decreased at first to 16% but decreased again and ended at 26%. In the weeks after the implementation period, the tags stayed present but slowly came off. Approximately 6 months later, three (10%) of the tags were still present. We observed throughout the feasibility test that the presentation of the area varied, which is to be expected in a busy supermarket. At times, the area looked messy; boxes would block access to some products, products would be sold out, some would change packaging, and new products would be introduced to the selection.

#### Customer responsiveness

When we asked customers about the tags, we learned that they had been unaware of them and that some believed that it was not something they would use—some did not know the meaning of the labels on the tags, while others did not find the labels relevant for them.

[The tags] don’t matter. My wife is pretty health conscious, so we don’t use those, let alone know with such a thing as breakfast cereal. (Male customer)

From our observations of the behaviour of the customers in the breakfast products and cereals department, we find two interesting groups: Those who shop alone and those who shop together with others (primarily children). These groups seem to practice different behaviours.

Among those who do their grocery shopping by themselves, we find two subgroups: 1) those who have planned or know exactly what they want to buy, and 2) those who decide at the store. For the first sub-group, we observed that some showed this by practising a behaviour where they would walk quickly and purposefully towards the shelves and quickly pick up a product. Others would look determined to find a specific product, as the fieldnote excerpt illustrates:

A woman stands looking at the muesli. She first grabs an orange bag on the bottom shelf, then a more yellow one next door and puts the first one back on the shelf. She inspects the bag she took. She starts to look around the shelves more and reaches for a bag that has a pinker look on the top shelf. She puts it back and reaches into the space next to it, where there are a few bags at the very back, but she has difficulty reaching them. A man comes by, notices the woman, and offers to help her. The woman indicates a yes, and the man reaches up and grabs a bag ‘that's the one!’ says the woman as the man hands her the bag.

Another example was a man who kept looking back and forth between some muesli and granola products and his phone before he eventually chose a product. It is unknown whether the man was looking at a specific note, a text request from his family, or a picture on his phone, yet what was on his phone seemed to determine the product he bought. Overall, this group seemed very unlikely to be influenced by the tags, as they had made their choice already before they entered the store.

For the second sub-group, those who seemed to make their decision in the store, we observed that some would just stop and glance at the products without choosing one before moving on with their shopping. Others would look more randomly at the selection than those described above, walk back and forth in the aisle, compare different products and read the info on the back of the products.

For those who shopped together with others (most often children), we observed that when adults shopped with children, the choices of the child and the choices of the adult often conflicted. In one example of a child and a woman who looked at breakfast cereal products, the child was initially allowed to pick a product and asked for different chocolate variants, which all featured cartoon figures; however, the woman rejected all of the child’s choices. In the interaction, the child was met with demands from the woman regarding the attributes of the products: they could not contain chocolate or sugar. In the end, it was the woman who chose a product based on her experience of the child’s preferences and her criteria. In similar situations, we did observe an attempt at compromising between the adult’s and the child’s criteria, which was explained by this woman:

I ask them [woman and boy aged about 10] what they look for when choosing breakfast cereals. The woman looks at the boy and says, ‘Well, what are we looking for?’. The boy does not answer but looks at her and me and smiles. The woman herself replies, ‘Something we can agree on. Something he likes but is not too unhealthy, either’. I ask her what she considers unhealthy. She waffles for a bit and then replies, ‘Yes, but he wants that Lions cereal, for example, and I don’t want him to have that. So something that’s not de facto sweets’. She takes the box of granola that they have chosen [Paulún's blueberry/lemon granola] out of the basket, looks at it and says, ‘So we chose this one. There's probably also a lot of fructose and caramelised stuff in it, but yeah.’

This illustrates the high impact children had on the choices of breakfast products, but also how the parents tried to control and negotiate the final choice.

#### Retailer perspective

The store manager had little faith in the effectiveness of the shelf tags:

The thing about tagging cereals, I don't think that makes the slightest difference. The reason why I’m sceptical in that regard is that it’s a mixture of what I do on a daily basis. It’s especially the behavioural patterns of our customers, but also how I act as a customer myself to a degree. I don't think shelf tags with the whole grain label or anything like that; in my experience it hasn’t changed things much. (Store manager)

His view on the effect of the initiative was in line with our observations of the customers in the store. Furthermore, the store manager explained that it was difficult to maintain the initiative, as it was not part of the employees’ daily routine. This was also the argument of why the tags lingered after the test period—it was just not part of the usual protocol either to hang them up or take them down. This perspective was shared by the føtex representative (B), who also highlighted the cost of this maintenance.

Contrary to the store managers’ sceptics, the føtex representative (B) was more positive about the initiative:

I think it’s a good initiative. We work a lot with tags and labels in general. [...] I think making it transparent to the consumer is really interesting because there’s nothing wrong with buying a box of Nesquick cereal every once in a while. At least we should not claim it’s the wrong thing to do. But you just have to be clear about what you’re buying, and I think those labels help with that. (føtex representative (B))

She explained that the initiative was highly compatible with their usual strategies. However, she also explained in the interview that a barrier to using shelf tags to promote the buying of certain products was that the chain was trying to reduce the printed material they used in their stores as part of their CSR strategy and to reduce costs.

### Replacement of the complimentary bun for children with a banana

The complimentary banana was fully implemented in the feasibility test period except for 1 day of observation, where the signs were not visible (Table 3). The initiative also remained available and present by the sign for at least 10 weeks after the implementation period. Furthermore, the store manager informed the researcher that they would continue to provide bananas for customers requesting this as an act of customer service. From the observations, we do find that the presentation of the initiative changed throughout the period. At first, the bananas were placed in a cardboard box on the display counter, which was later replaced with a nicer-looking basket. The number of bananas and their colour also fluctuated during the different days, which would be expected due to the delivery of the bananas and how often they are restocked. However, compared to the buns, we never observed that the bananas were not available, making it a reliable offer no matter the time of the day.

#### Customer responsiveness

We observed two ways (1 and 2) that the complimentary offer for children was brought up: 1) A customer would ask for the ‘bun for children’, or 2) the staff would offer the complimentary banana to buying customers. In the first way 1), we saw two responses from the staff (a and b) and the customers (i and ii): (a) The customer would be offered the bun with no mention of the banana, or (b) the staff would inform the customer that they no longer offered buns but that they offered a banana instead. The customers had two primary responses to this message: (i) The customer rejected the offer and decided to buy a bun or another item instead. The child was often included in this decision. (ii) The customer accepted the offer and received the banana. In some cases, the child did not accept the offer and the customer compensated for this response by buying a bun or another product for the child. In the second way 2), in which the staff offered the banana spontaneously, the customers almost always reacted positively and accepted the offer.

The following excerpt illustrates why some customers rejected the offer:

A woman with a child of about 1-year-old in a stroller walks up to the bakery and asks for a children's bun. The child has already noticed the buns from the moment they arrive and sits, pointing at the buns through the glass window and babbling. The shop assistant says that there are no children's buns but bananas and points to the sign. The woman replies, ‘I’d like to buy a bun, then’. The assistant takes the bun and enters it into the till, while the woman says, ‘Bananas are so messy’. The assistant smiles and says, ‘Well yeah, I'll pass that on’. The woman replies, ‘It's just that the banana is rather a bother, and the assistant replies, ‘But I think we’ll be offering [the buns] again eventually’.

Thus, adults rejected the offer because eating a banana was a messier process than eating a bun. During meetings and interviews, the retailer also highlighted this as the main reason for rejections of the offer, especially among those with younger children. Another reason for rejection was that the parents did not appreciate the offer nor perceived a need to offer their children a banana instead of a bun.

#### Retailer perspective

This initiative was the most successful and interesting one in the eyes of the store manager.

I’d like to highlight the banana for kids, which is clearly the initiative I found most customers were pleased with. (Store manager)

Many customers responded positively to the new offer, which was emphasised as a marker of success. It was also the reason why the initiative continued after the 6-week period, and the store manager explained that they would continue to give bananas to those who asked for them.

The following excerpt illustrates what the bun meant to føtex and the chain’s relationship with its customers.

The children's bun has been around for donkey’s years, and it’s become ingrained in parents and kids alike that you can get them in føtex. So, we’re quite interested in learning how many people would actually, if presented with the alternative, choose something else, like, for example, the banana. I’m quite surprised by that – we can't track it, unfortunately – but off the top of my head, up to 40 to 50 percent actually choose the banana. I find that very interesting. (føtex representative (B))

Thus, it came as a surprise that the initiative was so well received. However, despite the positive experiences with the initiative, the retailers also commented on the cost. They highlighted that the banana was more expensive than the bun, and if it should be an option offered in all stores, then it would have to be prioritised at the executive level as an additional expenditure. In this case, the banana would only be an alternative to the bun and not a replacement. This was rationalised by the retailers’ attitude of not making choices on behalf of the customers.

### Smaller bags for pick’n’ mix sweets

This initiative was not implemented until 8 weeks after the initial implementation date. It was fully implemented for five out of the six weeks; during the third week, we observed that the old, larger bags had been hung in front of the new smaller bags. At 2 weeks and four and a half months after the feasibility test, the smaller bags could still be found behind the larger bags—however, it is unlikely that these would have been used, as the obvious choice would have been the bag at the very front. As described for the other areas, this also fluctuated in its presentation and stocking.

#### Customer responsiveness

We did not get any direct reactions from customers on the smaller bag. However, our observations showed that different strategies were used to decide the amount of candy among customers who bought pick’n’mix sweets. Some showed signs of visually assessing the amount of sweets in the bag, which were the customers we would expect to influence. We often observed this strategy among adults with children, where it was the adult who would visually assess the amount and communicate to the child when they had picked enough.

Those with very young children would walk alongside the child and select the sweets for them, and some adults would encourage the choice of the child by pointing out different variants and commenting on the appearance of the sweets.

Other strategies were to mix according to a pre-defined number of pieces or volume:

A boy of about 10 and a girl of about 8 come over and mix sweets. They repeatedly weigh the bag while doing so. A woman comes over, and the girl says, ‘Hello mummy!’ The woman says, ‘Don’t forget to weigh it’. She then grabs a bag herself and begins to mix sweets. The boy asks the girl, ‘Did you weigh it?’. The girl walks over to the scales and says, ‘I think I’ve got enough’. However, she does not close the bag, and she begins to walk around somewhat restlessly, then says, ‘I don’t know what to pick. I’m still [a few] grammes short’.

An interesting aspect of the situation above is that the girl expressed that she was satisfied with what she had chosen, but she felt that she had to meet the prespecified weight and, therefore, tried to find more sweets to put in her bag. Such strategies undermine the mechanism which the initiative was trying to influence.

#### Retailer perspective

Overall, the retailers were positive about this initiative. The føtex representative (B) highlighted that this initiative was interesting as it was a stealth initiative, compared to the initiatives with the sodas, and would change the behaviour of the customers without them noticing. In her opinion, this was not a problem, as people paid per gram.

The store manager had a clear demand for the implementation; it should be easy for both the staff and customers to use. This perspective was backed up by a føtex representative (B) who said:

If there’s something that doesn’t work for us, it’s... if it doesn’t work for our customers, that’s what we need to solve first. (føtex representative (B))

This shows how one success criterion of the retailers is customer satisfaction, which we elaborate on later (See: Influence of customers and other stakeholders on store operation).

The initiative was very delayed, and one reason was that it was challenging to create a new bag that would work in the store. This resulted in the order of many different bags in large quantities due to the agreements with the suppliers, which had been very costly for the retailer.

The føtex representative (B) also reflected on what the potential evidence of an effect would mean to the retailer:

Then we’ll have to wait and see if people buy fewer sweets. And of course, this is something that we must take into account because it’s no secret that part of being a responsible business is to make a profit. And if we sell fewer sweets, then we make less money. (føtex representative (B))

This shows how health and financial profit were seen as opposites and how the success of the initiative would not necessarily lead to it being viewed favourably, as it would negatively affect their profit. Any implementation in the chain would, therefore, have to be a strategic decision.

### Facilitators and barriers

In the sections above, we have focused on the four specific initiatives. In the following, we will present analytical findings that go across the initiatives and elucidate what facilitated and hampered the implementation of the initiatives overall. We have organised our findings under three headings: Health is not the number one priority; General capacity of the retailer; and Influence of customers and other stakeholders on store operation.

#### Health is not the number one priority

In this section, we present the retailers’ motivation for and interest in engaging in the project and working with health and health promotion and what drives and/or curbs this motivation. In our understanding of motivation, we draw on Scaccia et al. [33] and view motivation as incentives and disincentives that contribute to the desirability of using an initiative focusing on health.

We find that the retailers expressed motivation for working with health and health promotion, which at first seemed to be based on interest. The retailer representatives explained how they personally were interested in health and wanted to learn more, but also that the organisation had an interest in health, especially among children and young people, and wanted to contribute to health-preventing activities, for example, by financially supporting local sports clubs. According to one retailer representative, this was because physical activity and healthy eating promote happier customers, as well as happy employees. The argument points to retailers’ focus on customer satisfaction (see: Influence of customers and other stakeholders on store operation). The focus on the customers relates to another factor of motivation: Working with health was also seen as a relative advantage in that customers increasingly demand healthier products and alternatives. Lastly, we found that the motivation for working with health was a feeling of obligation due to the view of having a social responsibility:

I would say, in purely business and commercial terms, we are, indeed, a commercial business that was created to make money. There’s no ignoring that (laughs). So, of course, this is our main KPI [key performance indicator]. But that being said, we also agree that we have a social responsibility because we are as big as we are. We make a lot of foodstuffs available to the Danes, as do many of our colleagues in our industry, so there is no doubt that we have a role to play in terms of what we make available. (føtex representative (A))

According to the excerpt, this obligation was rooted in the size of the organisation and, thereby, the major influence on people’s selection of food products. However, the excerpt also highlighted that health was not their first priority, which was profit. This point has been repeatedly mentioned among retailers, which reinforces its validity; they were a business and had to gain profit to keep running their operation, which presented limits for what could be implemented. The store manager even expressed how he perceived the running of a supermarket and promotion of public health as incompatible goals and something he had never seen an example of in a real-life supermarket.

However, from the interviews with the retailers and our fieldwork, it seemed that this was not completely black and white, as the retailers were willing to give up their profit in some cases. An example is the hiding of tobacco products in all Salling Groups’ supermarket chains, which they voluntarily implemented in 2018, which led to a significant decrease in profit from tobacco products.

After all, the Salling Group pioneered this with tobacco products. I'm proud of that, but I also think it’s the right thing to do. My personal opinion is that it was the absolutely correct move they chose to make, by making it harder to market a product that is obviously bad for my health. We’re not there with pick‘n’mix sweets just yet, in that we would claim they’re bad for your health, but the mindset in terms of; that is, upholding the mindset when it comes to cigarettes is something that we, as an industry, can easily support in close cooperation with, among others, yourselves [researchers] and the industry. (Store manager)

Risk seemed to be the driver. If the retailer was convinced that the risk was real or big enough, then they were willing to give up some of their profits because it was the ‘right thing to do’, and they would have the courage and power to do so. It was mentioned by all three informants that they did not believe in bans, limitations or hiding of products, as this interfered with the customer’s freedom of choice. This viewpoint was a barrier to the implementation of all initiatives that used strategies that would minimise or reduce the availability of a product. Yet, as with the tobacco products, we found other examples where this restriction of choice was justified by the retailer. One example was that the føtex chain only sold organic bananas. From a sign in the store, this was because:

‘we want to avoid the spray agent chlorpyrifos. Among other things, it is suspected of harming the development of children and foetuses. We can’t live with that suspicion and therefore you can only buy organic bananas in the future’

As with the cigarettes, the argument here was the health risks. In the interview with the store manager about restricting choices, animal welfare and political reasons (e.g. Russia’s warfare against Ukraine) were mentioned as other arguments for doing so.

So, despite an immediate motivation for working with health, the retailer also expressed how other interests and priorities may hinder and set aside the work with health.

#### General capacity of the retailer

This section presents our findings relating to the general capacity of the retailer in the form of resources, organisational size, and culture. General capacity is understood as the readiness or ability to implement any new initiative [33].

Through the interviews with the føtex representative (B) and working together with the retailer during the project, we have found that the retailer seemed to be used to and willing to implement new initiatives. In this current study, they accounted for all expenses related to the development of materials for the test and were also willing to risk some of their profit for a short period of time. The føtex representative (B) highlighted this high level of available resources several times in the interview:

I have some leverage, so when we do something, we don’t do it by halves. What I find most motivating, and I can say that with complete peace of mind, is that if the Salling Group says they’re going to do something, or if føtex says they’re going to do something or says they want to win this particular battle, then we win it, and then we do it to the full. [...] So when we say, for example, with this health project, that ‘we want to work with health,’ then we do want to work with health, and we’re going to make a difference in health, too. (føtex representative (B))

In this excerpt, she expressed that the mere size of the company allowed them to push any agenda if they wanted to. However, this also underlines that this capacity is dependent on the retailer’s willingness, a willingness that was not in favour of many of the initiatives that the researcher, based on the literature, thought would have the greatest effect.

Even though the size of the company came with many available resources, the retailer also explained how the size of the company had worked against the project in several ways:

What I think made it difficult for us to get through with some of these things let's just take the sodas, in that case, we have a private label collaborator who has production facilities, and when they press the ‘Salling sodas’ button, it doesn't just spew out a few thousand bottles, but millions. So saying ‘can't we just try to reduce the size and give it a try.’ It's a giant setup, so it’s not possible to do that at a whim. You’d need to get a whole or half chain on board that can help sell such volumes because otherwise, the costs would go through the roof. (føtex representative (A))

What this excerpt explains is that even changes that appeared small would take tremendous effort and be very costly, due to the size of the organisation.

Another challenge of the implementation was embedded in the retailers’ organisational culture. Føtex representative (B) explained in the interview that conflicting goals between employees made it difficult and time-consuming when implementing new initiatives. Another barrier to implementing the initiatives was high staff turnover at the retailer. In an interview with a føtex representative, she explained that people often shifted around different positions in the organisation, which ended in the project falling between two stools, leading to misunderstandings of agreements and changes in attitudes towards the initiatives.

In summary, we find that the retailers could, in some respects, have a strong general capacity to implement new initiatives by having available resources and being used to implement new initiatives. Regardless, this study shows that this was not utilised due to a lack of willingness. Moreover, we find that the size and organisational culture of the retailer hampered the implementation of the initiatives.

#### Influence of customers and other stakeholders on store operation

The last section reports on the influence of customers on the retailer’s willingness to implement the initiatives, and the influence of other stakeholders, especially producers, on what can be implemented.

We found that the customer’s reactions and attitudes were determining to the retailer when implementing any new initiative, as indicated in the sections above. According to the retailer, the customer was the focus when designing the layout of the store:

We are in very close dialogue with our clients, we do quantitative surveys and we do focus groups, we do in-depth interviews. And in that context, we're trying to understand, when you're shopping, how do you go about it. Is it easy for you to find the items you are looking for? And based on the responses, we try to adapt our stores to make things easy for our customers. (føtex representative (A))

The same representative also mentioned that she thought it would have been a strength of the project to have conducted interviews with the customers as a part of the development process, emphasising the weight they put on the customer’s attitudes. The retailers highlighted the importance of customer satisfaction and convenience in their shopping experience as a barrier to implementing certain initiatives, such as changing the placement of products. However, these same factors have also proven to be facilitators for other initiatives, such as the tags for breakfast products and the complimentary banana for children, as demonstrated above.

Another important stakeholder for the supermarkets was the suppliers of their products. Others were government actors (e.g. the Danish Veterinary and Food Administration). For both downsizing initiatives, the suppliers of the products (sodas and bags for sweets) were key to the success of their implementation. In an interview with the store manager, he explained the huge role some of these suppliers have in the daily operation of the store and the chain.

After all, we’ve got a chain agreement that our head office has made with the breweries. I don’t get to decide which items are in our refrigerators. [...] The tricky thing is that we’re not only dealing with føtex or the Salling Group. We also have to do with some other, equally large companies that are also just coming in. Plus, I have people here X times a week to service their particular area. [...] [Another thing] that proved tricky, as far as I recall, was that the alternatives offered, people felt strongly about those because the breweries made some strategic choices, and because of those, some of the items that we might be able to stock, they didn't want to sell those. (Store manager)

This excerpt illustrates how suppliers like the breweries, as shown earlier, influenced the implementation and affected the decisions made by the retailer.

This section indicates that even though the retailer is convinced that a given initiative would be interesting to implement in their supermarket, the suppliers often must agree as well, and finally, the customers must also welcome it.

## Discussion

In this study, we have explored the implementation, acceptability, and feasibility of four different health-promoting food retail initiatives aimed at customers in a real-life supermarket setting, using different qualitative methods. We found that (i) Two initiatives (downsizing of bags for the pick’n’ mix sweets and the complimentary banana for children) were implemented to a high degree, yet delivery issues caused delays according to the planned date, especially for the bags. The downsizing of soda bottles was not implemented as intended; the size and packaging deviated from the original plan due to delivery failure. Moreover, the implementation decreased over the feasibility test for the initiative with shelf tags, as it took more continuous maintenance. For all initiatives, we found that they lingered after the feasibility test; however, only the banana for children was somewhat sustained for a period to accommodate customer demand. (ii) The retailers expressed different levels of acceptability towards the initiatives, and different representatives sometimes also showed different levels of acceptability towards the same initiative, such as the tags on the breakfast products. The most well-received initiative was the banana for children, which is somewhat unsurprising, as it was the retailers themselves that suggested including this initiative. Additionally, the positive response from the customers that they got supported the retailers’ positive attitude towards the initiative. We also found that many customers responded well to this initiative; however, we also observed a group that did not accept the initiative and preferred the bun over the banana. For the remaining initiatives, customers did not seem to notice them. Yet, we did observe customer behaviours that would probably work against the suggested mechanisms of some of the initiatives. (iii) In general, we describe three themes of barriers and facilitators that influence the implementation and possible sustainment of the initiatives: Health is not the number one priority, General capacity of the retailer, and Influence of customers and other stakeholders on store operation. Firstly, we found the retailers were motivated to work with health, both from a personal and professional perspective. The motivation was rooted in a feeling of social responsibility as well as health initiatives being viewed as a relative advantage, due to demand and making customers happier. Still, other priorities, such as profit and maintaining customers’ ‘free choice’, challenged the motivation to implement such initiatives. Secondly, the retailer showed a high level of available resources, which supported their general capacity to implement the initiatives; however, the large size of the organisation and its culture proved to be barriers to the implementation. Lastly, the analysis showed that the influence of both customers and other stakeholders was crucial to the implementation, both in terms of what is possible and what the retailers would be interested in and prioritise.

Our findings are similar to those of others [26, 35]. Winkler et al. [35] found that even though supermarket actors found health-promoting initiatives meaningful to engage in, their engagement was challenged by a business mindset, practical routines, and structural requirements. Thus, despite the involvement of retailers in the development, selection and implementation of the initiatives, studies suggest that healthy food retail initiatives still encounter some fundamental barriers towards the implementation, such as the economical aspect or the view on customers’ free choice. However, our results also indicate that it might be possible to persuade food retailers to remove products or restrict choices if the evidence or arguments of it being the right thing to do are sufficiently strong, as with organic bananas or tobacco products. This has also been the case of another retailer in Denmark, which has decided that all their stores should be tobacco and nicotine-free by the end of 2028 to reduce the number of smokers [36]. Another solution is to identify win–win initiatives, as the complimentary banana for children was somewhat an example of (if we consider the banana as a healthier alternative) and which other studies have found as well [35, 37].

Even though the four initiatives were implemented (yet two not as intended) in this study, and we found them to be somewhat acceptable to the retailers, we must still highlight that these initiatives represent a very small portion of the initiatives first suggested and entail several compromises from what the researchers had initially planned (Duus et al. *Unpublished*). Moreover, the customer’s responses to the initiatives were mixed, and in some cases, their behaviour indicated that the initiatives would have little effect. Compared with studies testing similar initiatives, we find that 1) Shelf tags alone were found unlikely to change food purchases [38] and are likely to contribute to disparities in food purchases as not all customers know nutrition labels or have the literacy to read and understand them [39]. 2) Smaller bags for pick’n’ mix sweets could be successfully implemented and, based on results from another study, might be able to decrease the volume of sweets sold [40]. Moreover, others have also shown that customers are willing to buy smaller product options [41]. Taken together, this suggests that voluntary engagement with researchers might not suffice to make changes that would improve the supermarket environment as opted for to support population health. This view has also been suggested by Winkler et al. [35], and in the Lancet series on commercial determinants of health, an even more critical perspective on engagements with commercial actors as food retailers is presented [42, 43]. Here they warn against how commercial actors use partnerships with researchers, among others, as a tool to improve their reputation and credibility [42].

In our collaborative process with the retailer, we experienced many challenges. We did not accomplish aligning retailers’ and researchers’ interests as scholars have suggested being the prerequisite of implementing healthy food retail interventions in supermarkets [26, 27]. This underlines the importance of the pre-intervention phase, as described by Hawe, Shiell, and Riley [44], which is fundamental to a successful implementation. During the pre-intervention phase, the establishment of relationships between different people or agencies often occurs, and these relationships may play a crucial role in the implementation and the explanation of why some work and others do not [44]. In line with this, another study has suggested exploring what implementation strategies might promote the uptake of evidence-based interventions among food retailers [45]. They found that contrary to many other studies, the intervention in their study was compatible with the interest of the store managers to which it was presented—these store managers had a strong feeling of social responsibility towards the communities they operated in [45].

### Strength and limitations

The investigation of the feasibility test was strengthened by using different methods, process evaluation concepts, and a broad view including both the delivery and presentation of the initiatives as well as customer and retailer perspectives. We primarily got the retailer perspective from a strategic level, yet we had planned on conducting focus group interviews with staff at the test store to get perspectives from an operational level on the initiatives and the implementation process. However, no staff wanted to participate in an interview. The store manager explained that this probably was due to three things: 1) They had no interest in the study, or they were tired of the study, 2) the recruitment was done too late (approximately 2 months after the feasibility test period), and 3) the staff was overworked as a result of understaffing due to the coronavirus disease pandemic. Future studies aim also to analyse sales data in order to evaluate whether any changes in sales of the products we intervened on occurred. However, with the available data, we will not be able to analyse whether the initiatives change people’s eating patterns or whether they influence people differently in terms of their socioeconomic factors or other characteristics.

A thorough needs assessment [46] among supermarket customers to test the initiative’s assumptions and their food purchase patterns would have strengthened the study. However, this was not possible within the timeframe and funding scheme, so the development drew primarily on existing knowledge and the experience of the retailer and the Danish Cancer Society. Furthermore, the store visits conducted in the store during the development of the initiative also provided a few customer perspectives, which led to the exclusion of some ideas (Duus et al. *unpublished*).

Furthermore, we learned two methodological lessons from the in-store observations: 1) All observers were met by the feeling of being ‘in the way’ and a need to be in almost constant movement to not interfere with the order in the store. The observers were met with a feeling of self-awareness and a need to legitimise their presence at the store by wearing a sticker on their shirts saying ‘visitor’ or their university identification card. These feelings were amplified by the governmental advice of social distancing and the requirement to wear face masks in grocery stores, introduced during the period of observations. 2) Concerning this, the observers also found it challenging to approach customers for the short interviews due to the feeling of invading people’s private space, hence only five were conducted. This was especially challenging when wearing face masks, as it was impossible to produce and read non-verbal signals (e.g. smiles), and difficult to hear what people were saying.

### Implications for future studies and practice

This study presents an investigation of the implementation of healthy food retail initiatives for supermarkets that have been developed and selected together with retailers as suggested by the literature. It suggests that the implementation of such initiatives is possible and—to some degree—high. Yet, the quality of the initiatives was rather low, and some were not implemented as intended. Moreover, we still present some of the same barriers and limitations as former studies that have not implemented collaborative strategies in the pre-intervention phase. Some of this may be due to challenges such as a high staff turnover at the retailer and a lack of a shared understanding, as shown in another study (Duus et al. *unpublished*). Future studies must explore this further.

Lessons for future studies are to identify initiatives that customers appreciate, as this is important to retailers. Underlining a needs assessment as an important first step in intervention development [30, 46]. Furthermore, future studies should involve a broader range of stakeholders, including manufacturers and suppliers, in the development of the initiatives, as they have significant power over what can be implemented. Future studies would also benefit from identifying and testing implementation strategies that can facilitate the implementation of this type of intervention in this setting.

## Conclusions

We performed a qualitative investigation of the implementation, acceptability, and feasibility of four different healthy food retail initiatives aimed at customers in a real-life supermarket setting, which had been developed and selected together with retailers. Only two of the four initiatives were implemented as intended, and the perspectives of retailers and customers were mixed or unclear. Altogether, the study highlights the challenges of implementing healthy retail food initiatives despite early involvement of retailers in the selection and design of those initiatives. Adding to the challenges of implementation, the initiatives also represent a compromise between the interests of the researcher and the retailers and do not represent what the literature suggests as the most effective strategies. A compromise made to uphold the partnership and complete the funded research project. Future studies should further examine the impact and pitfalls of including retailers (or other commercial actors) in the development and selection of healthy food retail initiatives and try to identify successful implementation strategies facilitating implementation.

### Supplementary Information

Supplementary Material 1.

Supplementary Material 2.

## Data Availability

The data generated and analysed during the current study are not publicly available due to their sensitive and confidential nature but are available from the corresponding author upon reasonable request.

## Abbreviations

CSR: Corporate Social Responsibility
KPI: Key Performance Indicator
