# Supplementary material for: Implementation of health-promoting retail initiatives in the Healthier Choices in Supermarkets Study—qualitative perspectives from a feasibility study
Source: BMC Med. 2024 Sep 2;22:349. doi: 10.1186/s12916-024-03561-2 (PMC11367934; doi:10.1186/s12916-024-03561-2)
Supplement: Supplementary file 1 — Supplementary Material 1. [file 12916_2024_3561_MOESM1_ESM.docx]

**Additional file 1**

**Six key lessons from the pre-intervention observations in the test store**

1. **The route around the store**

Most customers followed a specific route through the store, which would take them to most of the areas of the store and guide them to the check-out desks (both self-administered and assisted). Customers who did not follow this route would stand out, they seemed to know the store well and go for a few specific items (e.g., lunch, cold drinks, and snacks). We also observed that adults with children would try to avoid the aisles with confectionary, snacks, and cakes. We also noticed that most customers would only do their shopping with one of the shopping baskets or just take the items they needed in their arms, those with baby strollers would also use them as a cart.

1. **The different times at the store**

The type of customers at the store and the flow of customers change during the day. In the morning and late evening, the pace is calmer and there are few customers, it is also mostly elderly people and adults with babies in the morning, and in the evening, it is mostly young people. Adults with children aged 5-12 were most often seen during the afternoon – we experienced seeing more women than men, the pace was picked up, and many more were shopping at this time. We did experience overall that many would do their shopping alone.

1. **Interactions with objects**

Many customers would practice a behaviour where they would stop and look at a product for a few seconds, they might pick it up and look at it and read on the back. Hereafter they either take it with them or put it back. Indicating a behaviour where decisions on what to do are made in the situation based on attributes of the product, its packaging or positioning. Many customers also use their phones during their grocery shopping, some would talk on the phone or do a video call, while others would just look at their phone a lot. It is suggested that many would have a grocery list on their phone, while we observed that others had a physical note in their hand as their grocery list.

1. **Interactions with children**

We observed that customers who did their grocery shopping accompanied by children had to deal with many interactions with them during this task. It spans from paying attention to a story about their day in kindergarten or what the child is experiencing in the situation right now, to asking or pestering for different items at the store, often confectionary, snacks, and cakes. The adults used many different strategies to handle the latter situation, some would give a simple rejection (“no”), others would start to negotiate the amount (“but only that one”, “just a small bag”) or the item (“what about this one?”), and others would accept the request (“okay”). Some adults also involve the children in grocery shopping either by asking them to help find specific items or asking them what to buy (e.g. “What would you like?” or “Should we buy some candy?”).

1. **Behavior of the staff:**

The employees were most visible in the store in the morning and the evening near opening and closing time, where they would stock and trim the store. During all times of the day, we would see how the staff would help customers find the items that they needed. The staff could also draw attention to a specific area (e.g., if the butcher were present, more customers would go look at the selection at the butcher desk), We also noted a very friendly tone between the staff members, especially the younger ones – they would chat to each other and talk about private matters.

1. **Sensory impression**

All observers noted how they were met by different sensory impressions at the store. It could be the smell of different foods, the sound of music or noise from the many people in the store or machines, or visual impressions from the use of colours, decors, or pictures.
