# Supplementary material for: Implementation of health-promoting retail initiatives in the Healthier Choices in Supermarkets Study—qualitative perspectives from a feasibility study
Source: BMC Med. 2024 Sep 2;22:349. doi: 10.1186/s12916-024-03561-2 (PMC11367934; doi:10.1186/s12916-024-03561-2)
Supplement: Supplementary file 2 — Supplementary Material 2. [file 12916_2024_3561_MOESM2_ESM.docx]

**Additional file 2**

**Observation guide for in-store observation during implementation**

Focus overall:

- Are the initiatives implemented and maintained as planned?
  - When are the shelves stocked, and who is responsible?
- Competing ‘initiatives’
  - What products are available/promoted close by the initiatives?
  - Are healthier products more expensive than unhealthy products?
- How are customers and employees responding to the initiatives/customers' interaction with the initiatives?
  - What type of customers respond to the initiatives (e.g. age, sex, families/singles)?
  - How is the customer flow, and when are the initiatives encountered? Do the flow/shopping patterns differ by time and weekday?
  - Do employees talk with the customers/colleagues about the initiatives?
- If possible, ask customers about their thoughts on the initiatives; have they noticed them? Do they find them useful/helpful?
  To what extent are you satisfied with...

…the supermarket environment and atmosphere?

…the supermarket layout and routing?

...the supermarket tidiness?

…the assortment of food products?

…the general product prices?

...the product discount prices?

Focus points for each initiative:

Promotion of healthier breakfast cereals and products using shelf tags

- Are the shelf tags placed correctly?
  - Are they present at all?
  - Are they placed with the right products?
- Do customers respond to the shelf tags?
  - Looking at the enhancement? Commenting on them?
  - Are children drawn to the enhancements?
  - Are customers aware of the meaning of the labelling (ask them)?

Downsizing of soda sold at the checkout desks

- Is the right size displayed?
- What other products are placed next to the sodas?
  - Does the cashier have a 500ml soda by their side?
- Do customers notice the smaller size?
  - Verbal comments, swapping to another product, running to the refrigerators with the 500ml bottles.
- How do the employees discuss the initiative with each other and customers?
  - Do they send customers to the bigger bottles if they complain?

Downsizing of bags for the pick ’n’ mix sweets

- Are the correct bags available?
  - Are other sizes available?
- Do customers notice the new bags?
  - Do they react to the size?
  - What are their reactions?
- Do customers tend to use several bags?

Replacement of a complimentary bun for children with a banana

- Are the bananas always an option/do they run out?
  - Ask staff: How many bananas/buns are given daily (approximately)?
- Are the staff aware of the new offer (free banana)?
- How do the staff in the bakery introduce the new offer to customers?
  - How is the new offer promoted in the store?
- How do parents and children react to the new offer?
  - Are the bananas making more of a mess than the bun (ask and observe)?
  - Ask parents: Why do you accept/deny the offer?
- How do the staff in the bakery handle unhappy parents and children?
